# Effects of Supervised Exercise-Based Telerehabilitation on Walk Test Performance and Quality of Life in Patients in India With Chronic Disease: Combatting Covid-19

**DOI:** 10.5195/ijt.2021.6349

**Published:** 2021-06-22

**Authors:** Jaini Patel, Barry A. Franklin, Disha Pujary, Gagandeep Kaur, Ankita Deodhar, Sakshi Kharbanda, Aashish Contractor

**Affiliations:** 1 Department of Rehabilitation and Sports Medicine, Sir H.N. Reliance Foundation Hospital and Research Center, Mumbai, India; 2 Department of Preventive Cardiology and Cardiac Rehabilitation, Beaumont Health, Royal OAK, Michigan, USA; 3 Department of Internal Medicine, Oakland University William Beaumont School of Medicine, Michigan, USA

**Keywords:** Chronic disease conditions, COVID-19, Health related quality of life (HRQoL), Physical fitness, Six-minute Walk Test, Telerehabilitation

## Abstract

**Background:**

The world is currently undergoing a pandemic, caused by the SARS-CoV-2 virus (COVID-19). According to the World Health Organization, patients with chronic illnesses appear to be at the highest risk for COVID-19 associated sequelae. Inability to participate in outpatient-based rehabilitation programs and being home-bound can increase the risk for and potential worsening of chronic health conditions. This study evaluated the short-term effects of telerehabilitation on patients' walk test performance and health related quality of life (HRQoL).

**Methods:**

47 patients (23 cardiovascular, 15 pulmonary, 9 oncology) participated in the telerehabilitation program. At baseline and following a 1-month intervention, patients had their 6-minute walk test distance (6MWTD) and HRQoL assessed. Average daily step counts were measured by the PACER App.

**Conclusions:**

Our results indicate that a short-term, supervised virtual telerehabilitation program had significant positive effects on 6MWTD and HRQoL in cardiac, pulmonary and oncology patients during COVID-19.

COVID-19 arrived as an unexpected, noxious and paralytic intruder in our lives. The impact of the associated pandemic on daily life has had far reaching consequences on healthcare, economic stability and social norms (Haleem et al., 2020). The world has adapted by modifying daily habits and services it took for granted only a few months earlier. The new lifestyle involves social distancing by largely remaining at home, working from home, and focusing on activities of daily living. Health care experts suggest that restrictions aimed at minimising the risk of local transmission of SARS-CoV-2 have led to increased sitting time and reductions in physical activity (Chen et al., 2020). According to data released by Fitbit in March 2020 (FitBit News, 2020), the pandemic has resulted in a significant global decline in physical activity levels (up to 24%) as compared with the same time last year. The actual decline could be far greater in countries like India where stricter governmental lockdown measures have been implemented. The associated activity restrictions and increased sedentary time are most likely to adversely affect older and unhealthy individuals with multiple co-morbidities.

According to World Health Organisation (WHO), Centers for Disease Control and Prevention-USA (CDC) and other health agencies, patients with chronic illnesses (e.g., cardiovascular, pulmonary, obesity, diabetes mellitus and oncology diseases) appear to be at the highest risk for COVID-19 associated sequelae (Chow et al., 2020; Garg at al., 2020). Accordingly, such patients are required to avoid outpatient rehabilitation (rehab) center visits to minimise the risk of exposure to the virus. Regular physical activity and structured exercise are considered safe and effective interventions in the primary and secondary prevention of chronic diseases (Durstine et al., 2013). Inability to participate in rehabilitation and being home-bound can increase the risk for and potential worsening of chronic health conditions due to increased sedentary behaviours, decreased physical activity levels and reduced daily energy expenditure (Chen et al., 2020).

In this dilemma between the heightened risk of contracting the virus versus the risk of exacerbating existing chronic illness, telerehabilitation appears to represent the most accessible and efficient intervention to maintain and enhance health and well-being. One systematic review and meta-analysis concluded that telemonitoring and telerehabilitation safely provided benefits similar to traditional care for cardiac patients (Chan et al., 2016). Institutes in the USA and Canada have published guidelines and resource materials for the practice and implementation of telerehabilitation for clinicians (Association & Manitoba, 2018; Pineau et al., 2006).

Regular physical activity and/or structured exercise has the greatest benefit on the least fit, least active population cohort (bottom 20%) (Chen et al., 2020; Durstine et al., 2013; Lavie et al., 2019). This study evaluated the effects of supervised exercise-based telerehabilitation using readily available monitoring techniques on patients' walk test performance and health related quality of life, employing a technology driven cardiac rehabilitation model as recently described (Babu et al., 2020).

## METHODS

The selection of our study population was conducted through convenience sampling during March and April 2020.

## PARTICIPANTS

Patients who were already participating in formal rehab at the center, with the latest visit not more than a month earlier, were approached for telerehabilitation. Our study population included 47 patients (23 cardiovascular, 15 pulmonary, 9 oncology [5 breast, 4 head and neck carcinoma]) who agreed to participate and enroll in the home-based intervention. Prior to beginning telerehabilitation, 45 patients had attended at least five outpatient rehab sessions and the remaining two patients had undergone a preliminary entry assessment.

## INCLUSION AND EXCLUSION CRITERIA

At least one in-person assessment session with a physical therapist was required for enrollment in the telerehabilitation program. Patients with cardiac, pulmonary or oncology conditions were included in the study population. American Heart Association (AHA), American College of Sports Medicine (ACSM), and American Cancer Society (ACS) guidelines for exercise testing and prescription were used to determine patients' clinical eligibility for participation. Patients with unstable vitals, known contraindications to exercise, recent symptoms, visual, cognitive or hearing impairment, and those high fall risk or requiring assistance with ambulation were excluded from participation. Patients were also required to be able to navigate the technology to receive a video call.

## PREPARATORY PHASE AND ENROLLMENT PROCESS

After obtaining written consent for enrollment, patients were stratified into 13 different groups based on their primary condition, frailty (special needs due to musculoskeletal and neurologic limitations, back pain, knee pain) functional status and availability. Each group included three to five patients. Patients were notified of their schedule through WhatsApp, and respective cohorts were formed. Thereafter, study subjects were given several habituation sessions to clarify the technical methodology of telerehabilitation. The program was modified over time according to the patient response and therapist feedback.

## REHABILITATION PROGRAM

The home-based exercise interventions were provided three days per week on JioMeet video conference or WhatsApp video calls. At the beginning of each session, vitals (pulse rate, blood pressure, SpO2 and/or blood glucose) were reviewed and recorded. Most patients (80%) had at least one monitoring device at home. Exercise programs were divided in three categories based on intensity: Light, Moderate or High, using AHA, ACSM and ACS guidelines for exercise testing and prescription. However, to maximize safety during exercise, the prescribed exercise heart rates were kept at least 10 beats below the 60-80% estimated maximum; rating of perceived exertion (RPE) was kept < 13 (“somewhat hard”) on the Borg scale and the lowest value of SpO2 permitted was 90%. Each exercise session on the video call lasted ~35 minutes. Exercise commenced with 5-10 minutes of warm-up using specific calisthenics for the disease condition (neck and shoulder flexibility exercise in cancer patients; lymphatic drainage exercise in breast cancer patients; breathing and chest expansion in pulmonary and open-heart surgery patients; and active mobility exercise in other cardiac patients). Aerobic exercise and strength training (20-25 minutes) were performed using different body movements, body weight or household items (e.g., water bottles). Patients were advised to complete a prescribed amount of brisk walking, approximating ≥ 30 minutes per session. Daily physical activity was recorded using a PACER pedometer app. All other consults, including medication review, weight management education, and psychological well-being, were conducted via telephonic conversations by the designated health care provider on a weekly or ad libitum basis.

## ASSESSMENT AND DATA COLLECTION

Outcome measures were recorded at baseline and following the telerehabilitation program. A 6-minute walk test (6MWT) was conducted virtually on all patients in the presence of a care giver at home and under continuous monitoring by a therapist on the video call. Patients were instructed “to walk comfortably for 6 minutes, keeping their RPE below 13 (Borg scale 6-20), corresponding to “fairly light” to “somewhat hard.” A PACER App was used to measure the distance walked because it was not feasible to have a straight walkway of ≥ 10 meters for all patients. Accordingly, patients were advised to walk continuously on an obstacle free path inside their home.

Health related quality of life (HRQoL) was obtained via the completion of questionnaires: Short Form – 36 (SF-36) for patients with cardiac conditions, Saint George Respiratory Questionnaire (SGRQ) for patients with pulmonary diseases, Functional Assessment of Chronic Illness Therapy – Breast +Lymphoedema (FACIT B+4) for patients with breast cancer, Functional Assessment of Chronic Illness Therapy – Head and Neck (FACIT H&N) for patients with head and neck cancer. Patients were sent the HRQoL scales and asked to accurately complete and return them. Those unable to complete the surveys were contacted by a therapist who assisted them in this regard.

Patients were educated on the use of a PACER App to monitor their daily step counts and advised to carry their phone during their walks. Weekly challenges were set up to establish walking step/distance targets. To provide positive reinforcement, study participants were able to view walking data for their counterparts, and a leader board of weekly champions. The average number of daily steps walked during the last week were recorded.

Patients’ outcome measures prior to telerehabilitation were retrieved from their previous records and documented as “Pre” and/or “Baseline data.” After one month of telerehabilitation, outcome measures were recorded under “Post” and/or “Tele” data.

## RESULTS

Of the study population (n = 47), 23, 15, and 9 had cardiovascular conditions, pulmonary disease and cancer, respectively. The mean ± SD age of all participants was 61.2 ± 12.5 years. Patients with pulmonary disease averaged 68.2 ± 11.6 years, those with cancer averaged 53.9 ± 10.2 years and study participants with cardiac disease were in the mid-range, 59.6 ± 12.0 years. Overall, there was equal distribution of patients based on sex (men = 24, women = 23). However, cardiac conditions were predominant in men (n = 18), whereas pulmonary disease was more prevalent in women (n = 11). Related demographics regarding the primary diagnosis of patients’ conditions are shown in Table 1.

**Table 1:** Distribution of Patients According to Chronic Disease Condition

Chronic Disease Conditions	
Cardiovascular Diseases	
PTCA	6 (26%)
CABG	10 (43%)
Primary Prevention	3 (13%)
Cardiomyopathy	2 (9%)
Others	2 (9%)
Pulmonary Diseases	
Interstitial Lung Disease	8 (53%)
Asthma	4 (27%)
Emphysema	2 (13%)
Others	1 (7%)
Oncology Diseases	
Breast Cancer	5 (55%)
Head and Neck Cancer	4 (45%)

CABG = Coronary artery bypass grafting

PTCA = Percutaneous transluminal coronary angioplasty

Table 2 shows 6-minute walk test distance (6MWTD) results before and after the telerehabilitation intervention and the average number of daily steps walked during the last week of the program. Overall, there was a statistically significant 13.9% improvement in the 6MWTD after one month of telerehabilitation. Relative 6MWTD improvements were 17%, 9% and 12% for cardiac, pulmonary and oncology patients, respectively. The average number of steps per day by patients was 4097 during last week of telerehabilitation. Cardiac patients walked the highest number of steps per day, 5341, whereas cancer patients walked the least, 2195.

**Table 2:** Six Minute Walk Test Distance and Average Daily Step Count

	Six Minute Walk Test distance (in meters)		Average daily steps for last week
	Pre Mean ± SD	Post Mean ± SDlast week	
Overall	381.0 ± 137.8	433.5 ± 163.3	4096.8
Cardiac	444.8 ± 122.8	521.0 ± 113.6	5340.9
Pulmonary	308.9 ± 137.2	336.6 ± 175.2	3302.5
Oncology	333 ± 99.4	372.5 ± 145.5	2195.3

Pre = Pre telerehabilitation data; Post = Post telerehabilitation data

HRQoL as measured by the SF-36 showed no diminution during the telerehabilitation program, despite the strict lockdown. HRQoL assessments for pulmonary patients using SGRQ showed statistically significant declines in symptoms and disease impact. There were 51.3%, 44.9% and 29.0% decreases in the symptom score, impact score and total scores, respectively, following telerehabilitation. Trial Outcome Index, calculated by subtracting social and emotional components from the FACIT total scores, revealed a statistically significant 9.0% improvement in the post telerehabilitation scores for all oncology patients (Table 3). Parametric data were analysed using paired t tests and non-parametric using Wilcoxon matched pair signed rank tests. Table 4 describes statistically significant outcome measures after 1-month of the telerehabilitation program.

**Table 3:** Health Related Quality of Life Measures

	Pre	Post
*Short Form – 36*		
n	9	19
Score	76.9 ± 12.2	85.9 ± 13.3
*Saint George Respiratory Questionnaire (SGRQ)*		
n	14	14
Symptom Score	45.8 ± 22.5	22.3 ± 15.9
Activity Score	68.8 ± 22.7	61.1 ± 14.4
Impact Score	32.9 ± 20.0	18.1 ± 12.7
Total Score	46.1 ± 19.0	32.8 ± 12.8
*Functional Assessment of Chronic Illness Therapy (FACIT)*		
n	6	8
Total Score	131.8 ± 13.3	141 ± 10.4
Trial Outcome Index	85.7 ± 12.3	93.4 ± 8.2
Fatigue	39.2 ± 5.5	45 ± 5.7

Pre = Pre telerehabilitation data; Post = Post telerehabilitation data; TOI = Trial Outcome Index (Sum of Physical, Functional, and additional components of FACIT Scale).

**Table 4:** Statistically Significant Parameters

	Pre	Post	Difference	% Change	p Value
6MWTD (meters)	381.0	427.4	+ 35.0	**+ 9.2**	0.0418
SGRQ Symptom Score	45.8	22.3	-23.5	**-51.3%**	0.0025
SGRQ Impact Score	32.9	18.2	-14.8	**-44.9%**	0.0078
SGRQ Total Score	46.1	32.8	-13.4	**-29.0**	0.0067
FACIT-TOI	85.7	93.4	+7.7	**+9.0**	0.0313

6MWTD = Six Minute Walk Test Distance; FACIT= Functional Assessment of Chronic Illness Therapy; Pre = Pre telerehabilitation data; Post = Post telerehabilitation data; t-paired t test; SGRQ= Saint George Respiratory Questionnaire; TOI= Trial Outcome Index (Sum of Physical, Functional and Additional components of FACIT scale); w-Wilcoxon matched pair signed rank test; p < 0.05 is statistically significant.

## DISCUSSION

Daily step count provides a readily accessible tool to monitor and establish physical activity goals. Recent evidence suggests an inverse dose–response relationship between daily steps and health outcomes, including all-cause mortality, cardiovascular events, and type 2 diabetes (Kraus et al., 2019). According to a recent report (Tison et al., 2020), due to COVID-19 the average number of steps walked per day has markedly decreased. The fall is most dramatic in countries where governments have implemented strict housing lockdowns, except to purchase essentials, social distancing, and non-essential business closings. Similar changes were seen in India. According to a real time step count data released by GoQii activity tracker of over 5 lakh users in India, the average daily step count fell from 6432 to 3146, between March 2 to March 17, 2020. The associated 51% reduction was a week before complete lockdown in the country was announced (Singal, 2020), after which the step counts likely fell even further.

In 2017, investigators reported on daily step recordings of 717,527 individuals from 111 countries for 68 million days. Across the globe, individuals walked an average of 4961 steps per day while in India the figure approximated 4297 steps (Althoff et al., 2017). The present findings suggest that our patients (n = 47) walked an average of 4097 steps each day at the end of the telerehabilitation intervention. Although data prior to the telerehabilitation program are lacking, these values are comparable to average daily steps walked by individuals in India, despite the complete lockdown.

In 2016, a study conducted in Denmark for patients participating in a cardiac telerehabilitation program reported a mean ± SD of 5899 ± 3274 steps per day (Thorup et al., 2016). Our cardiac patients walked an average of 5341 ± 3790 steps per day which is comparable. The average daily step count for our patients with pulmonary conditions was 3303 ± 2989, which was considerably lower than their cardiac counterparts. This finding may be due to multiple factors, including their older age, a greater number of female participants, and their relatively lower pulmonary reserve (5 patients required supplemental oxygen during activity). Moreover, the average daily step count for our oncology patients was even lower, 2195 ± 668 steps per day. Their severe ambulatory impairment is likely attributed to the debilitating effects of cancer and its treatment. In addition, inaccurate step counts due to slower walking speeds cannot be discounted (Beevi et al., 2015; Kuys et al., 2014).

A relevant Cochrane review (Anderson, 2017) of 43 studies compared the effects of home versus center-based rehabilitation. These data and other reports (Piotrowicz et al., 2010; Scalvini et al., 2013), found that home-based and hospital- or center-based cardiac rehabilitation appear to be of comparable effectiveness in improving clinical and HRQoL outcomes in cardiac patients. Similarly, the present study found a statistically significant 13.9% improvement in 6MWTD after telerehabilitation in all disease conditions. Relative improvements were 17.1%, 9.0% and 11.9% for cardiac, pulmonary and oncology patients, respectively.

Although the HRQoL of our cardiac patients (SF-36) improved by 11.6%, it was not statistically significant, due to small and unequal sample sizes of pre versus post telerehabilitation data. However, we had complete data on HRQoL for pulmonary (SGRQ) and oncology (FACIT) patients which revealed statistically significant improvements, 29% and 9%, respectively. The 51.3% decrease in symptoms (SGRQ/score) in our pulmonary patients may be attributed to additional effects beyond exercise. Air pollution is a major public health problem affecting 90% of individuals living in urban areas worldwide, which negatively impacts patients with asthma, chronic obstructive pulmonary disease, lung cancer and respiratory infection (Jiang et al., 2016; Kurt et al., 2016). According to recent studies (Mahato et al., 2020; Sharma et al., 2020), the air-quality levels of Mumbai improved by 32% (32% decline in AQI) as compared with the previous year because of a significant decline in the number of vehicles allowed on the road. This may have spuriously amplified the positive effects of telerehabilitation on the symptom scores of our patients.

Contemporary communication and technology tools can be used to enhance health behaviours, including regular physical activity and structured exercise (Douma et al., 2018; Tate et al., 2015). However, the maximum benefit is achieved when the activity tracking is combined with weekly educational sessions and/or specific challenges. Another study in medical students found that the addition of weekly emails and group challenges combined with an activity tracker (Fitbit) was more effective in increasing step counts compared with an activity tracker alone (7429 ± 2833 vs. 6483 ± 2359) (DiFrancisco-Donoghuea et al., 2018). Integration of the PACER App and interactive chatting groups for our patients proved to be effective in promoting goal-setting and regular physical activity, encouraging self-monitoring and providing feedback and social support.

We also found better short-term adherence of patients who enrolled in home-based telerehabilitation as compared with group-based outpatient rehabilitation, and this was consistent across all chronic disease conditions. Similar findings of higher adherence with home-based rehabilitation have been previously reported (Piotrowicz et al., 2010). Current evidence indicates that the adherence of oncology patients to outpatient-based rehabilitation remains a challenge for varied reasons. Based on our experience, the adherence of cardiac and pulmonary patients to outpatient rehabilitation varies between 90% and 95%. In contrast, for patients undergoing cancer treatment, adherence is ~ 84% and 62% for breast and head and neck cancer, respectively. Achieving 100% adherence for these patients with a 1-month home-based telerehabilitation program is an important finding in the field of oncology rehabilitation and has the potential to bring about a paradigm shift or hybrid model in the treatment of cancer patients.

In closing, perhaps Dr. Ken Powell summed it up best when he stated: “Some activity is better than none, and more is better than less” (Zhu, 2019, p. 525). Our unique findings suggest that a short-term telerehabilitation program can improve HRQoL and help to combat the hypokinetic consequences of COVID in varied patient subsets with chronic disease.

## BARRIERS TO ENROLLMENT

Our patients reported some barriers to enrollment and participation in the telerehabilitation program. Some missed the opportunity to use the exercise equipment provided in the outpatient department, especially the treadmill and stationary cycle ergometer. Others had poor data network and connectivity issues and/or were uncomfortable using technology. Interestingly, few patients reported the reason for their dissatisfaction as not having direct (in-person) interaction with staff. These barriers are similar to those described in a previously published systematic review (Kruse et al., 2018).

## PATIENT ACCEPTANCE

Our experience confirmed that patients who enrolled and participated in telerehabilitation enjoyed it. Most found it more accessible than outpatient-based rehabilitation, and after just 1 week of therapy, none of the patients had any difficulties operating the video call for exercise or PACER App. In addition, they demonstrated greater adherence as compared with conventional outpatient-based rehabilitation.

These findings are supported by another study that evaluated pulmonary patients’ attitudes towards telerehabilitation. Although some patients were indifferent, others were reassured by the surveillance and safety that the virtual format provided, as well as the motivation and structured regimentation for physical training (Dinesen et al., 2013).

## LIMITATIONS

Small sample size is one of several limitations inherent in our study methodology. Certain assessments also lacked adequate baseline data. Another limitation of our study was the inability to directly conduct physical assessments on telerehabilitation program participants. Developing modified virtual assessment techniques and test protocols that provide more validity and reliability would be helpful in this regard. Moreover, the reliability of self-measurement techniques, equipment used at home, and the pedometer App may not match the preciseness of traditional outcome assessments. However, frequent education and practice on measurement techniques and the development and use of rehab specific tools may help to enhance quality and reliability.

To reduce the potential for exercise-related acute cardiac events at home, only low risk patients were selected for participation. In addition, we monitored for adverse symptoms, reduced the intensity of exercise as compared with our traditional outpatient-based program, used conservative criteria for RPE and allowable SpO2 decreases for pulmonary patients, and provided education on management of emergencies at home. The absence of any significant exercise-related complications suggests that this methodologic approach to telerehabilitation is justified. In fact, only one patient complained of back pain related to pre-existing lumbar spondylosis. Nevertheless, the relatively small number of subjects (n = 47) and short duration of the program (1 month) precludes definitive conclusions regarding the safety of the telerehabilitation program.

## FUTURE PERSPECTIVES

A larger sample size and longer telerehabilitation intervention (e.g., 3–6 months) should allow future investigators the opportunity to further clarify the safety and effectiveness of such programs. Utilization of other features of the App to monitor activity time, distance covered, weight and waist circumference could also be used to provide additional outcomes. Development of comprehensive platforms to help administer virtual treatment options (e.g., history taking, assessment, informed written consent, fitness testing, exercise prescription, and documentation) may improve the efficiency of telerehabilitation and the preference for telerehabilitation by patients as well as their healthcare providers. Telerehabilitation could emerge as an attractive alternative for selected patients (e.g., oncology) undergoing treatment and for those for whom outpatient-based rehab may not be feasible.

## CONCLUSION

Our results indicate that a short-term, supervised virtual telerehabilitation program has significant positive effects on walk test performance, daily ambulation and HRQoL in cardiac, pulmonary, and oncology patients. Use of a step counter with regular education in home-based interventions could enhance health by promoting regular physical activity even during strict home lockdowns, thereby preventing exposure to new viral mutations.

## ABBREVIATIONS

ACS: – American Cancer Society
ACSM: – American College of Sports Medicine
AHA: – American Heart Association
CABG: – Coronary Artery Bypass Grafting
CDC: – Center for Disease Control and Prevention-USA
CL: – Confidence Limit
COVID-19: – Corona Virus Disease 2019
DASH: – Disability of Arm and Shoulder Scale
FACIT: – Functional Assessment of Chronic Illness Therapy: 5 components: Physical, Social, Emotional, Functional and additional concerns
FACIT: B+4 – Functional Assessment of Chronic Illness Therapy – Breast Cancer + Lymphoedema
FACIT-F: – Functional Assessment of Chronic Illness Therapy – Fatigue
FACIT H&N: – Functional Assessment of Chronic Illness Therapy – Head and Neck Cancer
TOI: – Trial Outcome Index; Sum of Physical, Functional and Additional components of FACIT Scale
HRQoL: – Health Related Quality of Life
PTCA: – Percutaneous Transluminal Coronary Angiography
RPE: – Rate of Perceived Exertion
SD: – Standard Deviation
SF-36: – Short form – 36 scale
SGRQ: – Saint George Respiratory Questionnaire
SpO2: – Oxygen Saturation
WHO: –World Health Organisation (WHO)
6MWT: – Six Minute Walk Test
6MWTD: – Six Minute Walk Test Distance
